# Efficacy of Core-Strengthening and Intensive Dynamic Back Exercises on Pain, Core Muscle Endurance, and Functional Disability in Patients with Chronic Non-Specific Low Back Pain: A Randomized Comparative Study

**DOI:** 10.3390/jcm13020475

**Published:** 2024-01-15

**Authors:** Raee Saeed Alqhtani, Hashim Ahmed, Hussain Saleh H. Ghulam, Abdullah Mohammed Alyami, Yousef Hamad Hassan Al Sharyah, Reyaz Ahmed, Ashfaque Khan, Abdur Raheem Khan

**Affiliations:** 1 Physiotherapy Program, Department of Medical Rehabilitation Sciences, College of Applied Medical Sciences, Najran University, Najran 61441, Saudi Arabia; rsalhyani@nu.edu.sa (R.S.A.); hsghulam@nu.edu.sa (H.S.H.G.); rahmad@nu.edu.sa (R.A.); 2 Department of Physiotherapy, Integral University, Lucknow 226026, India; diriiahsr@iul.ac.in (A.K.);

**Keywords:** core strengthening exercise, intensive dynamic back exercise, pain, functional disability, endurance

## Abstract

Background: Chronic back pains are progressively disabling working individuals, including 60–80% of the general population, for which their diagnosis is challenging to healthcare workers worldwide, thereby becoming a burden to nations. Purpose: The study aimed to investigate the efficacy of core strengthening exercise (CSE) and intensive dynamic back exercise (IDBE) on pain, core muscle endurance, and functional disability in patients with chronic non-specific low back pain (LBP). Methods: The study was based on a three-arm parallel-group randomized control design. Forty-five participants with chronic non-specific LBP were recruited and randomly divided into the CSE, IDBE, and Control groups. The CSE and IDBE groups received CSE and IDBE, respectively. However, the Control group received no intervention. Numeric pain rating scale, Oswestry Disability Index, core flexors, extensors, and side bridge tests assessed pain intensity, functional disability, and endurance of core muscles. Outcome scores for the dependent variables were collected at baseline (pre-intervention) and six-week post-intervention. There were no follow-up measurements in this study. A one-way multivariate analysis of covariance (MANCOVA) was used to analyze the intervention effects on the outcomes within groups and between groups, respectively; keeping the significance-level alpha at 95%, i.e., *p* < 0.05. A univariate F-test was performed to observe the superiority of one treatment over another. Pearson’s correlation coefficient test was conducted to determine a relation between the dependent variables. In all statistical analyses, the level of significance α was kept at 0.05. Results: All forty-five out of sixty-three participants with chronic non-specific low back pain (male, 32 and female, 23; average age, 20.24 ± 1.46 years; average pain duration, 19.6 ± 5.42 weeks) completed the study and their data were analyzed. The MANCOVA test showed a significant difference between the treatment groups on the combined multiple endurance tests for the core muscles (flexors, extensors, side bridge tests to the right and left), Visual Analog Scale (VAS), and Oswestry Disability Index (ODI) scores after controlling for baseline scores of all the dependent variables: F (6, 12) = 23.381; *p* < 0.05; Wilks’ Λ = 0.033; partial η^2^ = 0.819. A post hoc pair-wise comparison followed by a univariate F-test indicated that a significant improvement was found between the CSE vs. IDBE vs. Control groups on the post-test scores of all the dependent variables except VAS and EET (CSE vs. IDBE only). A Pearson’s correlation coefficient test revealed a notable relation between the dependent variables. Conclusions: The experimental group CSE was found to be more effective than IDBE on improving functional disability, cores’ flexors, and side bridges’ endurance tests than IDBE. The magnitude of this improvement exceeded the minimal clinically important difference (MCID), suggesting a clinically relevant enhancement in functional disability, core flexors, and side bridge endurance for participants engaged in CSE. However, CSE vs. IDBE revealed non-significant differences on reducing pain and core extensors’ endurance. The absence of statistically significant differences suggests that the observed changes did not exceed the established MCID for pain intensity and core extensors’ endurance. In addition, partial eta-squared value revealed the superiority of CSE over IDBE and Control groups. This suggests that the observed differences between the two interventions are not only statistically significant, but also clinically relevant, surpassing the established MCID.

## 1. Introduction

Low back pain (LBP) is a global health concern affecting approximately 80% of individuals at some point, ranking as the leading cause of worldwide disability [1]. It significantly impacts individual well-being, manifesting in a reduced quality of life, impaired workability, and elevated healthcare costs for those seeking multidisciplinary spinal care compared to primary care [2]. LBP has diverse causes, including muscle strain, ligamentous sprains, herniated discs, and degenerative spine changes, often influenced by risk factors like age, sedentary lifestyle, poor posture, and obesity. Acknowledged as a complex issue with biopsychosocial dimensions, LBP involves psychological and social elements such as stress, anxiety, and work-related factors [3,4]. If not effectively managed, LBP can lead to complications like chronic pain, decreased mobility, and a decline in overall quality of life [5].

The comprehensive management of LBP Involves a holistic approach, integrating pharmacological and non-pharmacological strategies per guidelines [6]. Physical exercise is a consistently recommended intervention for non-specific LBP, emphasizing early initiation of non-pharmacological treatments, including patient education, self-management, addressing the psychosocial aspects of pain, and the resumption of normal activities and exercise [7,8].

The primary goal of physical treatments is to improve function and prevent disability [9]. Existing evidence allows for flexibility in exercise participation, whether in group or individual programs, as no superiority of one type over another has been established [9]. Active strategies, particularly exercise, correlate positively with decreased disability [8]. Conversely, passive methods like rest and medications are discouraged due to their association with worsening disability, making them not recommended for LBP management [9].

Guidelines from Denmark, Belgium, the United States of America, and the United Kingdom uniformly endorse exercise as a standalone or combined intervention with other non-pharmacological therapies [10,11,12]. These multidisciplinary treatments, including tai chi, yoga, massage, and spinal manipulation, acknowledge diverse approaches beneficial for LBP patients [6]. Public health programs are encouraged to play a crucial role in educating the public about preventing LBP, focusing on lifestyle modifications, ergonomic practices, and the importance of regular physical activity [12,13].

The existing literature highlights positive outcomes associated with various exercise modalities, such as core strengthening exercise (CSE) and intensive dynamic back exercise (IDBE). The increasing popularity of integrating CSE into LBP rehabilitation is driven by documented changes in abdominal muscle activation patterns associated with LBP [13,14]. CSE benefits include decreased pain, an alleviation of movement phobia, improved joint proprioception, static and dynamic balance, and enhanced muscle thickness in patients with subacute non-specific LBP [13]. Moreover, CSE shows effectiveness in addressing excessive lumbar vertebrae translation and rotation in patients with non-specific chronic LBP due to lumbar segmental instability [14].

As advancements in training techniques and classification approaches in physical therapy for LBP continue, comprehensive research is increasingly recognized as imperative. While studies have reported the individual effectiveness of CSE and IDBE, a notable gap exists: no studies have directly compared the efficacy of core-strengthening and intensive dynamic back exercises in managing chronic, non-specific low back pain. Therefore, this study aimed to determine and compare the effectiveness of core-strengthening and intensive dynamic back exercises on pain intensity, functional disability, and muscle endurance in patients with chronic, non-specific low back pain. The study hypothesized that there will be a significant difference between the effect of CSE and IDBE on pain intensity, functional disability, and muscle endurance in patients with chronic, non-specific low back pain. The study will determine a more suitable and effective exercise protocol between the two therapeutic approaches, and will also provide evidence-based guidance for physiotherapists rather than relying on a trial-and-error method for selecting exercises from a multitude of options.

## 2. Materials and Methods

Study Design: The study employed a three-arm parallel-group randomized superiority trial design, focusing on comparative interventions to assess their effects on key outcomes, including pain, muscle endurance, and functional disability, at baseline and after the completion of six-week interventions. This study design did not include follow-up assessments for the outcome variables.

Ethical Consideration: Ethical approval was obtained from the Ethical Committee of Integral University under reference no. IE/IIMS&R/2022/43, dated: 15 December 2022, aligning with human rights protection and ethical research practices. The study adhered to the Declarations of Helsinki (2013) and the ethical guidelines of the study country. The trial for this study was officially registered online at ClinicalTrial.gov Protocol Registration System with the trial ID NCT05708781 dated: 31 January 2023. There were no changes with reference to the RCT protocol after trial commencement. Every participant provided a signed informed consent form, as evidence of their voluntary agreement to participate in the research.

Sample Size: The effective sample size was determined based on a pilot study comprising 15 participants (5 from each group) to ensure study power. Pain intensity, as a primary outcome variable score was considered for the analysis. A priori calculations, including computerized ANOVA tests, set parameters such as power (1-β) = 80%, significance level (α) = 0.05, mean differences = 3.57 ± 1.31, allocation ratio N1/N2 = 1, and effect size = 0.63. This led to an effective sample of forty-five participants (15 in each group). Factoring in a 20% attrition rate, a total of fifty-four participants were required for the study.

Study Setting: The orthopedic surgeon assessed morbidities through a combination of clinical examinations, medical tests, and patient-reported outcomes. The evaluations were conducted by the same orthopedic surgeon and his associates and referred to the outpatient department, Physiotherapy, at the medical facility of Integral University, Lucknow, India for obtaining physiotherapy interventions. The recruitment spanned eleven months, from December 2022 to November 2023, with a convenience sampling method utilized for participant screening based on eligibility criteria.

Participants: Forty-five participants with chronic non-specific low back pain meeting the study’s inclusion and exclusion criteria were recruited. Chronic non-specific low back pain (LBP) is defined as a persistent and long-term discomfort or pain in the lower back region that lacks a specific identifiable cause or pathology. This type of low back pain is characterized by its duration, lasting for a period of at least 12 weeks, and the absence of clear underlying structural or anatomical abnormalities that can explain the pain [15,16]. Inclusion criteria encompassed males aged 25–55 with chronic non-specific low back pain lasting at least 12 weeks, and the absence of clear underlying structural or anatomical abnormalities that can explain the pain, while the exclusion criteria included severe or radiating pain, specific medical conditions, mental illness, and other factors potentially interfering with training or cooperation. The recruitment followed a first-come-first-serve approach until the required sample size was achieved.

Outcomes: Two assistant physiotherapists administered the outcome measures and they were kept blinded to group allocation. They evaluated three dependent variables—pain intensity, functional disability, and core muscle endurance—utilizing the Visual Analog Scale (VAS), Oswestry Disability Index (ODI), and various core muscle endurance tests. The VAS, a subjective pain rating scale, is reliable and valid. It covers a range of 10 cm, ranging from zero (indicating no pain) to ten (representing extremely unbearable pain). For patients with subacute or chronic low back pain, the minimal clinically important change (MCIC) for pain on a Visual Analog Scale (VAS) should at least be 20 mm [17]. In adherence to instructions, participants marked their pain intensity on the scale using a pencil, with values between 0 and 10 [18,19]. Oswestry Disability Index (ODI)—a reliable and valid functional disability outcome tool—is a self-administered questionnaire that lists activities susceptible to compromise due to low back pain [19,20]. ODI showed a good internal consistency (Cronbach’s *α =* 0.85), whereby standardized regression weights is relatively high for all ODI items (0.5–0.7), and discriminative ability of ODI is superior at higher levels of disability [21,22]. MCIC for functional disability on ODI was found to be at least 10 points [17]. Conforming to the guidelines outlined by McGill et al. [23], four fundamental endurance assessments were executed. The aim of these tests was to maintain a static position for the maximum duration. These assessments encompassed the trunk flexor endurance test, trunk extensor (Sorenson) endurance test, and bilateral side bridge tests [24,25]. This comprehensive approach aimed to thoroughly assess pain, functional disability, and muscle endurance in participants with low back pain. The intra-rater reliability (ICC = 0.87–97), minimum detectable changes (15.02–24.14), and standard error of measurements (SEM = 5.42–8.71) for all core endurance tests were proven to be high [26], and scored zero (0) for MCIC [27].

Flexors Endurance Test (FET)

Trunk support was at a 60° flexion angle. The knees and hips were flexed at 90°, arms crossed over the chest, and feet securely positioned. Subsequently, the trunk support was withdrawn, and the participants endeavored to sustain this position for the maximum duration achievable. The test concluded when each participant could no longer maintain their position [24,25].

Extensors Endurance Test (EET)

Participants laid down in a prone position on a treatment table with their lower body parts fixated, including pelvis, hip, knees, and ankle, while the upper half of their bodies, including trunk chest, and their heads were supported with a leveled stool with wheels, which was subsequently moved away at the start of the test. Participants lifted themselves from their position until their torsos were parallel to the floor with their upper limbs across their chests to rest on opposite shoulders. Further, they were instructed to maintain this horizontal position for as long as possible, the failure of which concluded the test. The endurance time was measured with a stopwatch, beginning when the participants assumed the horizontal position and concluding when there was a loss of control in the test position [24,25].

Bilateral Side Bridge Test (SBT)

For this test procedure, participants were directed to extend their legs while lying on their sides on a treatment couch, placing the top foot in front of the lower foot for support. Participants were distributing their body weight on their elbows and feet while their hips were lifted off the surface. The test concluded when the participants were unable to hold the straight-line position and their hips dropped and made contact with the supporting surface. The same test procedure is repeated for the opposite side [24,25].

For all the outcome variables, baseline (pre-intervention) and post-intervention assessments were obtained on day one and after completion of six weeks of intervention. The follow-up assessment for the outcome variables were not included in this study.

Procedures: Forty-five patients with chronic non-specific low back pain were recruited in the study based on the inclusion and exclusion criteria. After meeting the eligibility criteria, participants were thoroughly briefed on the study’s objectives and procedures, and written informed consent was obtained from each participant at the study’s outset. Subsequently, all participants were randomly assigned to one of three equal groups (*n* = 15 per group): CSE, IDBE, and Control. To prevent or minimize the participants’ requests from the risk of crossover and potential recruitment bias, a robust randomization process was conducted through the online website http://www.randomization.com (accessed on 19 December 2022), and a simple random sampling method was employed, followed by proper monitoring and a follow up of the participant’s specified intervention. To maintain participant’s privacy and anonymity, at the reception counter, a concealed envelope containing the respective participant’s serial number and corresponding group name was distributed, with the participant’s serial number clearly marked.

Measurements for all outcomes were taken at baseline by an assistant physiotherapist who remained unaware of the participants’ group allocations. Specialist physiotherapists administered specified physical therapy interventions to participants in their respective groups, including CSE and IDBE interventions for the CSE and IDBE groups, and a standardized intervention common to all groups, including the control group. In this study, the physical therapists remained blinded, a condition facilitated by their non-author status in the ongoing research. They were aware of the study’s existence but were kept unaware of group allocations (experimental or control) because participant assignment to therapists was under the control of the department head. The therapists were instructed to administer specified interventions without inquiring about the participants’ identities or reasons behind the interventions.

**Figure 1:**
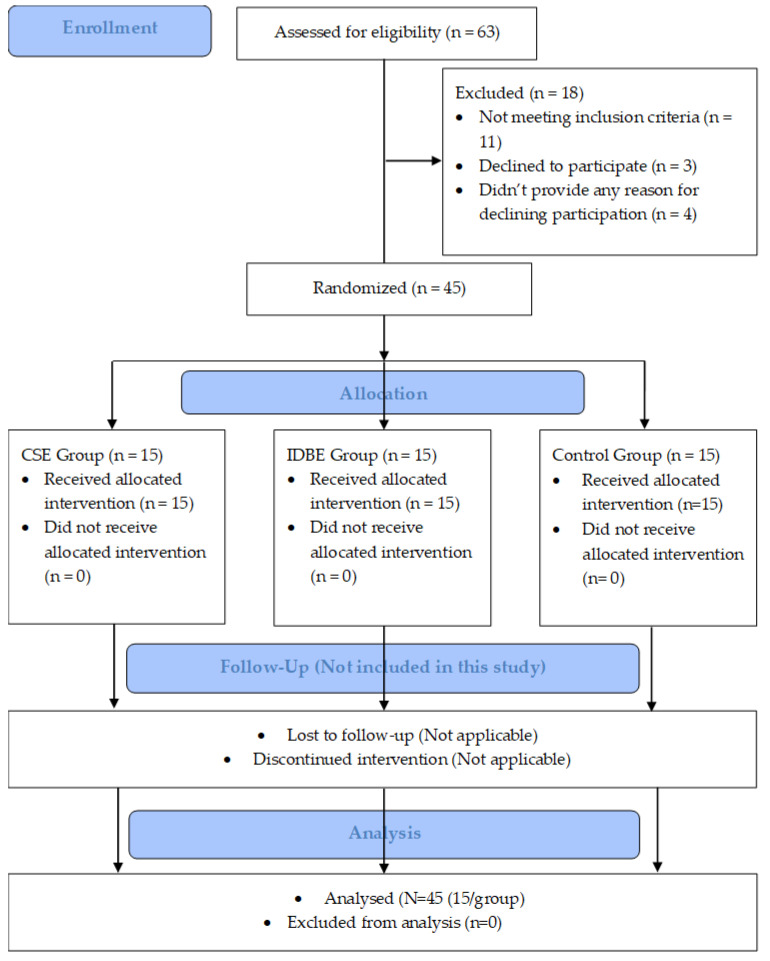
A CONSORT (2010) flow diagram depicts the study procedures, including participants’ recruitment, randomization, intervention group allocation, and data analysis. CSE: core-strengthening exercise; IDBE: intensive dynamic back exercises.

Post-intervention assessments were conducted by the same assessor after the conclusion of six weeks of interventions. However, no follow-up assessments for the outcome variables were scheduled in this study. The study’s procedural details, including participant enrolment, randomization, interventions, and analysis, are visually presented in Figure 1 of the CONSORT (2010) flow diagram.

Interventions

The Control group received the standard intervention, which is usually given a basic treatment, including hot pack (temperature at 70–80°; time = 20–30 min/session), active stretching (slow sustained stretching for 3–5 repetitions/session, hold time = 20–30 s, 30 s gap between two consecutive sets), and isometric exercise (isometric contraction hold time = 10 s; 10 repetitions/a set of 3 sets/sessions; 1 min gap between two consecutive sets), while the CSE and IDBE groups received the standard intervention plus CSE [12,13,14] and IDBE [26,28], respectively. The specialist physiotherapists delivered the stipulated intervention to their respective groups. Their expertise was judged based on their highest academic qualifications and specialized training certificates. In addition, feedback from the head of the department about their skills and experience was taken before the start of the study. The therapists used a unified intervention regimen to limit the administered intervention’s variability. The physical therapists and head of the department assessed for potential harm through patients’ feedback, physical examinations for red-flag signs, specific tests related to the discomfort/outcomes from any patient complaint, and follow-up assessments. The participants were controlled and closely monitor when undertaking any other physical therapy intervention or drug during the study periods.

CSE Group: Core Strengthening Exercise (CSE)

Isolated lumbar stabilization training

The core strengthening exercise (CSE) group engaged in a comprehensive program targeting isolated lumbar stabilization muscle training. This entailed developing an increased awareness of specific isometric contractions targeting stabilizing muscles. Participants concentrated on activating the transverse abdominis muscle by performing exercises such as hollowing the lower abdomen from both 4-point kneeling and lying positions. In a similar vein, engagement of the multifidus muscle was achieved through activities like stepping while standing, lifting the contralateral arm, and sensing the contraction of the opposite-side multifidus muscle. Emphasis was placed on precisely repeating isolated isometric co-contractions, involving both the transverse abdominis and multifidus muscles simultaneously, whether from sitting or standing positions. The program also integrated lumbar-stabilizing muscle activity into light functional tasks, stressing the importance of maintaining neutral lumbopelvic postures, isolating movement in adjacent body areas, and sustaining lumbar spine stability. Participants practiced controlling both neutral lumbopelvic postures and potentially aggravating postures, incorporating exercises such as hip horizontal abduction, heel slides, and leg slides from a crook lying position. Intensity began with gentle movements and progressed based on comfort; frequency was 3 alternate days weekly for six weeks and the duration was 5–7 min per session.

Bridge Track (Floor and Physio-Ball Routine)

In the Bridge Track routine, both on the floor and utilizing a physio-ball, patients follow a structured sequence of movements to enhance lumbopelvic motion and promote core strengthening and stability. The patient begins by exploring lumbopelvic motion, actively seeking a neutral lumbar spine, and engaging the abdominal muscles. Subsequently, an active gluteal contraction is initiated as the patient raises the pelvis to its maximum height while maintaining a neutral spine. These fundamental movements can be seamlessly incorporated into various bridge variations, ensuring a comprehensive approach to lumbar stabilization. The included variations encompass floor bridge, ball bridge, floor bridge marching, ball bridge with a single leg, bridge with feet on the ball, and hamstring bridge with feet on the ball, providing a diverse and progressively challenging set of exercises for patients in the program. Intensity began with 2 sets of 5–7 repetitions per side and progressed as tolerated; frequency was 3 alternate days weekly for six weeks and a duration of 7–10 min per session.

Dead Bug Track (Floor and Physio-Ball Routine)

The Dead Bug Track, designed for both floor and physio-ball exercises, focuses on abdominal training with a specific emphasis on maintaining spinal stability while integrating movements of the extremities. The exercise begins in a supine position with knees flexed and arms at the sides. Progression involves coordinated arm and leg movements in opposite and alternating motions, emphasizing diagonal isometric holds to target oblique abdominal muscles. Intensity began with 2 sets of 5–7 repetitions per side and progressed gradually; frequency was 3 alternate days weekly for six weeks and duration was 7–10 min per session.

Quadruped Track (Floor Routine)

The Quadruped Track, conducted on the floor, incorporates alternate arm and leg movements to enhance core strengthening and stability. In the quadruped position, where hands align under the shoulders and knees under the hips, the routine includes variations such as alternating leg movements with a stable trunk and opposite arm and leg (cross crawl) movements, ensuring a comprehensive approach to core strengthening and stability. Intensity started with 2 sets of 5–7 repetitions per side; frequency was 3 alternate days weekly for six weeks and duration was 7–10 min per session.

Supine Track (Physio-Ball Routines)

The Supine Track involves physio-ball routines designed to enhance core strength and stability. The patient initiates the exercise in a spine hook-lying position with feet on the ground and hips and knees moderately flexed. Throughout the routine, the arms can be positioned in increasing levels of difficulty, including reaching forward, crossed over the chest, crossed behind the head, and elbows extended overhead. The concentric phase of the crunch is considered complete when the inferior angle of the scapula lifts off the floor or ball. Variations within this track include the center Crunch ball and oblique Crunch ball, providing a structured and progressive approach to supine exercises using a physio-ball. Intensity began with 2 sets of 5 repetitions; frequency was 3 alternate days weekly for six weeks and duration was 5–7 min per session.

Side-Support Track (Floor Routine)

The Side-Support Track, a floor routine, is specifically designed to effectively target the quadratus lumborum muscle. Key technique points within this routine include maintaining a slight extension in the hip and ensuring that the shoulders, knees, and ankles form a straight line. The elbow is flexed at a 90-degree angle and is directly positioned under the shoulder for optimal form. The pelvis is then raised until the spine achieves a straight alignment. Variations in this track include the Side-Support with knees flexed at 90 degrees and Side-Support with knees fully extended, providing a focused and progressive approach to training the quadratus lumborum muscle. Intensity started with 2 sets of 5 repetitions per side; frequency was 3 alternate days weekly for six weeks and duration was 5 min per session.

Prone Track (Physio-Ball Routine)

In the Prone Track, part of the physio-ball routine, the patient initiates the sequence with feet against a wall for support, knees flexed and in contact with the floor, and subsequently extending the legs while leaning over the ball. As the ball rolls forward, emphasis is placed on straightening the spine to a neutral position, avoiding hyperextension. The initial exercise in this track focuses on enhancing the static endurance of the multifidus muscle, incorporating increased hold times. Starting with the arms by the side, progression then includes moving the arms to 120 degrees of abduction. The second exercise within this track shifts the focus to phasic control, introducing repetitions to train the superficial erector spinae muscles, particularly when the arms are positioned in front of the chest in a crossed manner. Variations in this track include Superman over the ball (with arms at 90 degrees of abduction) and repetitive spine extensions over the ball, providing a comprehensive approach to multifidus muscle training. Intensity started with 2 sets of 5 repetitions; frequency was 3 alternate days weekly for six weeks and duration was 5–7 min per session.

Standing Track (Physio-Ball Routines)

The Standing Track, part of the physio-ball routines, is designed to concurrently train the quadriceps and gluteal muscles while instructing the patient on maintaining lumbar lordosis during squatting and lifting activities. The physio-ball is strategically positioned in the small of the back, with feet placed hip-width apart and the body leaning over the ball to facilitate proper lower extremity alignment. The initial exercises focus on mastering a shallow squat, gradually progressing to a full squat with thighs parallel to the floor. The side squat variation places emphasis on the gluteus medius, with the pelvis slightly dropped on the inside leg, followed by pressing into the ball and leveling the pelvis to the maximum recruitment of the gluteus medius on the stance side. The final standing exercise involves lunges, where precise lower extremity alignment is critical. Both forward and back legs maintain alignment, with variations, including the full ball squat, single-leg ball squat, side ball squat, and static lunges, offering a diverse and comprehensive approach to quadriceps and gluteal muscle training. Intensity started with 2 sets of 5 repetitions; frequency was 3 alternate days weekly for six weeks and duration was 5–7 min per session.

IDBE Group: Intensive Dynamic Back Exercise

The intensive dynamic back exercise, as outlined in previous studies [26,28], comprises the three key exercises below.

Trunk lifting

Perform the exercise while lying face down on a couch, positioning the hips at the edge. The upper body remains unrestricted but is supported by the hands against the floor. Secure a strap over the calves, and lift the trunk with hands on the forehead, extending to the maximum capacity in the hips and spine. Initial support from a physical therapist may be necessary, and during pauses, a chair in front of the couch aids patient stability.

Leg lifting

It is performed by standing at the edge of the couch, leaning forward into a prone position with hips against the edge, flexed at 90 degrees, knees at 45 degrees, and feet on the floor. Secure a strap over the chest, and use straps around the knees to keep the legs aligned. Extend and lift both legs to their maximum.

Pull to neck

The procedure is performed from a seated position on a stool, with arms extended and spread above the head, gripping a weight lever. Using sub-maximal resistance, the lever is drawn behind the neck and shoulders.

The exercise regimen consists of 10 repetitions with a 1 min break between each attempt. The first and second exercises are repeated 50 times, and the third exercise is performed 50 times as well. This cycle is repeated twice, with a 15 min break, during which a hot pack is applied. Consequently, each of the three exercises is executed 100 times as part of the back program.

To address muscle soreness and post-training discomfort, each session concludes with a thorough stretching of the involved muscle groups. Initially, the physically demanding training commenced with a graduated approach, wherein patients performed each of the three exercises 50 times in the first session. Subsequently, in the following sessions, the repetitions increased to 60 times, and this progression continued until the completion of the full training in the maximum course.

Statistical analysis

The data analysis employed the statistical software SPSS (V.25, IBM Corp., Armonk, NY, USA) to assess the impact of interventions on dependent variables at baseline and after the end of six-week interventions without follow-up. Normal distribution for continuous variables, including VAS, ODI, and multiple endurance tests for the muscle groups of cores, was examined using a Shapiro–Wilk test. A one-way multivariate analysis of covariance (MANCOVA) was employed to test whether the multiple endurance tests for core muscles (tests for flexors, extensors, and side bridges to the right and left), the Visual Analog Scale (VAS), and the Oswestry Disability Index (ODI) scores differed by treatment (CSE, IDBE, and Control), after controlling for baseline scores of all the dependent variables. In addition, the univariate analysis of covariance (ANCOVA) was conducted to examine the effect of independent variables on post-test scores for different dependent variables, including VAS, ODI, FET, EET, SBTLt, and SBTRt [29]. Furthermore, Pearson’s correlation coefficient identified correlations among all outcome measures. The significance level (α) for all analyses was set at 0.05.

## 3. Results

Out of the initial sixty-three participants with chronic non-specific low back pain, forty-five (male, 32 and female, 23; average age, 20.24 ± 1.46 years; average pain duration, 19.6 ± 5.42 weeks) were assessed for the recruitment in this study. Among them, eleven participants did not meet the inclusion criteria, three declined to participate in the study due to unavailability, and four left without providing a reason after the assessment session was completed. Out of 45 participants, 2 females (mean age 23.5 years) and 1 male (24 years) were married, 5 were graduated and employed, 34 were employed (male 27 and female 13) after completion of senior secondary level (10 + 2 level), 6 were studying and unemployed, and 33 were smokers (male 29 and female 4). Eleven were weekly involved in sports activities, 29 were not on any regular physical activity and sometimes play football or indoor games, and 15 were not doing any physical activity. None of them were taking any regular medication for their back pain except paracetamol occasionally.

Test of normality

Each participant successfully underwent their designated six-week intervention, and the reported zero compliance rate underscores the goodness of a well-conducted trial. The baseline analysis revealed an overall homogeneous distribution among groups, indicating insignificant differences in demographic characteristics. The Shapiro–Wilk test for normality indicated an overall normal distribution of all outcome measures within each group, with insignificant differences for demographic characteristics, except for BMI (Kg/m^2^) in IDBE (*p* = 0.024) and Control (*p* = 0.046) groups, as outlined in Table 1.

Multivariate Analysis of Covariance (MANCOVA)

An in-depth multivariate analysis of covariance (MANCOVA) test after controlling for baseline scores, revealed a significant difference between the treatment groups on the combined dependent variables, such as multiple endurance tests for the core muscles (tests for core’s flexors, extensors, and side bridging muscles to the right and left), Visual Analog Scale (VAS), and Oswestry Disability Index (ODI) scores after controlling for baseline scores of all the dependent variables, F (6, 12) = 23.381, *p* < 0.05, Wilks’ Λ = 0.033, partial η^2^ = 0.819, indicating the intervention’s large effect size meticulously outlined in Table 2.

The group explained a substantial amount of variance in the combined dependent variables, with a very strong association. Follow-up univariate ANCOVA indicate statistically significant differences in post-test scores for all dependent variables (VAS, ODI, FET, EET, SBTLt, and SBTRt). The *p*-values for each variable are less than the conventional significance level of 0.05, suggesting that the differences observed are unlikely due to chance.

Furthermore, the effect sizes (η^2^) for each dependent variable are substantial, ranging from 0.681 to 0.915. This suggests that the independent variables have a considerable impact on the observed variations in post-test scores. Overall, the findings support the conclusion that there are significant differences in the post-test scores among the levels of the independent variables for each dependent variable, as indicated by the ANCOVA results.

Between-Group: Pairwise Comparison

At six-week post-intervention, the pairwise comparison of all outcome variables between treatment groups revealed significant differences between CSE vs. IDBE (*p* < 0.05) except for the variables pain and core extensors endurance (*p* > 0.05), CSE vs. Control (*p* < 0.05), and CSE and IDBE groups demonstrated significant differences compared to the Control group (*p* < 0.05), as presented in Table 3. A minimal clinically important difference was also achieved for all the variables. The detected discrepancy exceeds the predetermined minimal clinically important difference (MCID), signifying a clinically significant improvement in various aspects of functional ability for individuals engaged in core-strengthening exercises (CSEs), including functional disability, core flexors, and side bridges endurance. In contrast, the lack of statistical significance may suggest that, despite improvements, the observed changes did not reach the established MCID for pain reduction.

A Bonferroni adjustment for multiple comparisons was applied, with statistical significance accepted at *p* < 0.005 (0.05/6 dependent variables). All reported *p*-values were below this threshold, confirming the robustness of the findings (Table 3).

Additionally, the correlation between all the outcome scores at baseline (pre-intervention day 1) was established using the Pearson correlation coefficient test. All the outcome variables showed moderate-to-high correlation with each other; however, VAS showed a strong positive correlation with ODI (r = 0.937; *p* = 0.001), as described in Table 4.

## 4. Discussion

Involving 45 participants, this research conducted a comparative analysis of core-strengthening and intensive dynamic extension exercises in individuals with chronic LBP. The presentation of data remained consistent and uniform across all three groups. The analysis of this study’s baseline and 6-week post-intervention scores yielded compelling findings, indicating substantial variations in all outcome measures across each group. In addition, a minimal clinically important difference was also achieved for the variables ODI, core flexors, and side bridges endurance. These results strongly suggest that the implemented intervention holds considerable promise in inducing positive changes within the studied population, thus supporting its overall effectiveness. Notably, a post hoc test designed to discern the intervention effect between treatment groups revealed significant differences between the core-strengthening exercise (CSE) and intensive dynamic back exercise (IDBE) groups at the 6-week post-intervention assessment for all the dependent variables, except VAS and EET. Moreover, the significant distinctions were evident when comparing the CSE versus Control and the IDBE versus Control and the CSE groups over the IDBE and Control groups.

These differential outcomes underscore the nuanced impact of the interventions, suggesting that the CSE significantly differs in its efficacy with IDBE, both of which are notably more effective than the absence of structured interventions represented by the Control group. This underscores the importance of considering the intervention’s absolute impact and its relative effectiveness compared to non-intervention conditions.

Moreover, the exploration of the magnitude or size of the intervention effect, assessed through a partial eta-squared test, also revealed the superiority of the CSE group over the IDBE group. This nuanced perspective on the effect size contributes valuable insights into the comparative effectiveness of these interventions, indicating that although both are effective compared to control, CSE may have a larger effect size over IDBE.

The findings of the current study are in line with the previous studies, which revealed the effectiveness of CSEs in improving pain and functional limitations in patients with chronic LBP [12,13,14]. Other studies revealed the IDBE’s effectiveness as well, closely aligned with current study findings in terms of relieving pain, improving functional status, and enhancing muscle strength and core endurance [26,28]. The CSE’s and IDBE’s effectiveness is evident in patients with chronic LBP because they work through some basic principles that lead to changes in outcome scores once employed.

Core-strengthening exercise (CSE) effectively reduces pain, improves functional disability, and enhances muscle endurance in chronic LBP by impacting physiological and biomechanical processes. CSE enhances lumbar spine stability, positively affecting pain by strengthening core muscles, including the transversus abdominis and multifidus. This strengthens the muscles around the core, improving spinal alignment and reducing load on lumbar discs [30]. Increased core strength improves proprioception and postural control, alleviating lower back strain and pain. CSE enhances functional abilities by targeting coordination, balance, and neuromuscular control through specific core exercises [31]. Focused on improving endurance, CSE enhances overall physical performance, reducing fatigue during tasks [32].

The IDBE offers pain reduction through mechanisms such as improved blood flow and tissue healing. Dynamic movements enhance flexibility, reduce muscle stiffness, and alleviate chronic low back pain [33]. These exercises engage multiple muscle groups, promoting coordinated movements and enhancing overall functional capacity [34]. IDBEs improve joint stability, neuromuscular control, and endurance, addressing functional limitations associated with chronic low back pain. Involving repeated movements, these exercises boost muscle endurance over time. Adapting and strengthening back muscles through dynamic exercises contribute to enhanced endurance, providing better spine support during daily activities [35].

Recognizing the biopsychosocial aspects alongside identifiable causes and risk factors is crucial for developing comprehensive prevention and treatment strategies [5,6]. A thorough screening for ‘red flags’ during patient evaluations is recommended to exclude potential serious pathologies, and diagnostic tests, including imaging, should be performed if suspicion arises [7]. Additionally, assessing psychosocial risk factors, termed ‘yellow flags’ in prognostic screening tools, aims to predict and identify factors associated with less favorable outcomes, guiding a more comprehensive patient management approach [6,9]. Guidelines advocate integrating psychological programs for individuals with persistent symptoms to address the psychosocial aspects of pain [7,12].

In considering these findings, it is essential to acknowledge the multifaceted nature of chronic LBP and the potential influence of individual differences. Additionally, the study’s time frame may warrant consideration, as longer-term follow ups could provide a more comprehensive understanding of the sustainability of the observed effects. These findings collectively contribute to the ongoing discourse on intervention strategies for chronic low back pain and emphasize the need for tailored approaches based on both efficacy and individual patient characteristics. Further research with extended follow-up periods and larger sample sizes may refine our understanding and provide more nuanced insights into the comparative effectiveness of these interventions.

Clinical Significance

The results of this study carry significant implications for the clinical management of chronic LBP. CSE intervention has proven a greater effectiveness than IDBE in reducing functional disability and improving core flexors’ and side bridges’ muscle endurance. However, CSE has the advantage of better improving the functional disability, cores’ flexors, and side bridges to the left and right muscles’ endurances than IDBE. However, the selection between these interventions may hinge on diverse factors, encompassing patient preferences, resource availability, and clinical feasibility and judgment.

Notably, the Control group also exhibited improvements in certain outcomes, albeit with smaller effect sizes. This emphasizes the necessity of considering the natural progression of chronic LBP and the potential for spontaneous recovery. Nevertheless, structured interventions like CSE and IDBE seem to provide additional benefits beyond what might naturally occur.

The generalizability of the study findings extends to the broader applicability of core-strengthening exercises (CSEs) as an effective intervention compared to in-depth body exercises (IDBEs) in specific aspects of rehabilitation. The observed effectiveness of CSE in reducing functional disability and enhancing core flexors’ and side bridges’ muscle endurance suggests its potential utility across diverse populations with non-specific low back pain. Furthermore, the study’s findings indicate that CSE and IDBE exhibit comparable efficacy in reducing pain levels and improving core extensor endurance. This suggests that both interventions may be equally effective in addressing these particular aspects of non-specific low back pain. The partial eta-squared value, revealing the superiority of CSE over both IDBE and the Control group, adds to the generalizability of the findings. This statistical measure emphasizes not only the statistical significance, but also the clinical relevance of the observed differences. However, it is crucial to consider variations in patient characteristics, intervention implementation, and contextual factors when applying these findings to different populations and clinical settings. Further research across diverse cohorts is recommended to enhance the robustness and generalizability of the study outcomes.

Study limitations and future recommendations

Despite the valuable insights gained, this study has noteworthy limitations. The limited sample size and the lack of extended follow-up data might constrain the applicability of the results. In addition, in a rehab study containing the patients with chronic back pain, the sample size estimated was calculated, choosing pain as primary outcome instead of a measure of disability. A minimal clinically important difference was found to be zero (0) for the outcomes of the core muscles’ endurance tests [34]. Moreover, the study did not explore potential interactions between interventions and individual patient characteristics, and a comprehensive patient analysis of classification into stability and instability groups was proven to be unfeasible. The reliance on subjective tests to measure isometric endurance introduces inherent limitations. Muscle recruitment confirmation solely through palpation adds another layer of constraint. Subjects’ inability to be excluded from practice/training sessions, coupled with active engagement in regular competitions, may have introduced confounding factors affecting the study’s outcomes. Looking forward, addressing these limitations necessitates future research with larger and more diverse samples, extended follow-up periods, and subgroup analyses. This approach could offer a more nuanced understanding of the long-term effectiveness of interventions and enable tailoring treatment approaches to individual needs. Additionally, future investigations should extend beyond non-specific low back pain cases to encompass individuals with segmental instability, incorporating objective methods like stabilizer biofeedback, scanner ultrasound, and electromyography to confirm muscle recruitment. The incorporation of isokinetic testing overcomes the subjective nature of isometric endurance testing, ensuring a more precise and objective analysis of training-induced changes in trunk muscles. Extending the study duration to at least six weeks and broadening the scope to include female subjects would further enhance the generalizability of the results.

## 5. Conclusions

In conclusion, the experimental group CSE was found to be more effective than IDBE on improving functional disability, core flexors’, and side bridges’ endurance tests than IDBE. The magnitude of this improvement exceeded the minimal clinically important difference (MCID), suggesting a clinically relevant enhancement in functional disability, core flexors’ and side bridges’ endurance for participants engaged in CSE. However, CSE vs. IDBE revealed non-significant differences on reducing pain and cores’ extensors endurance. The absence of statistically significant differences suggests that the observed changes did not exceed the established MCID for pain intensity and core extensors’ endurance. In addition, partial eta-squared value revealed the superiority of CSE over IDBE and Control groups. This suggests that the observed differences between the two interventions are not only statistically significant, but also clinically relevant, surpassing the established MCID. This nuanced understanding provides valuable insights for physiotherapists, offering evidence-based guidance in the nuanced management of chronic non-specific low back pain. Identifying the superior efficacy of core-strengthening exercises (CSEs) underscores the importance of tailoring interventions to achieve optimal outcomes for patients undergoing rehabilitation for non-specific low back pain.

## Figures and Tables

**Table 1:** Showing the participants’ demographic characteristics, baseline scores for the outcomes, and test for normality using the Shapiro–Wilk test (95% CI for mean, *N* = 45; Missing = 0).

Variables	Groups (n = 15/Group)	Baseline Scores (Mean ± SD)	Shapiro–Wilk Test of Normality (95% CI)		
			Statistics	df	p-Value
Age (years)	CSE	20.47 ± 1.41	0.922	15	0.207
	IDBE	19.93 ± 1.22	0.897	15	0.087
	Control	20.33 ± 1.76	0.927	15	0.250
Height (cm)	CSE	174.00 ± 5.28	0.897	15	0.086
	IDBE	173.67 ± 4.88	0.936	15	0.329
	Control	173.20 ± 5.91	0.891	15	0.070
Weight (Kg)	CSE	79.93 ± 11.30	0.923	15	0.215
	IDBE	72.27 ± 10.41	0.907	15	0.122
	Control	74.07 ± 11.07	0.883	15	0.053
BMI (Kg/m^2^)	CSE	26.40 ± 3.45	0.910	15	0.137
	IDBE	23.93 ± 3.06	0.860	15	0.024
	Control	24.67 ± 3.10	0.879	15	0.046
VAS (cm)	CSE	6.00 ± 1.20	0.918	15	0.181
	IDBE	5.93 ± 1.16	0.931	15	0.278
	Control	6.00 ± 1.07	0.934	15	0.316
ODI	CSE	63.07 ± 9.59	0.972	15	0.883
	IDBE	60.27 ± 8.92	0.944	15	0.434
	Control	63.60 ± 10.60	0.943	15	0.417
FET	CSE	57.40 ± 23.03	0.954	15	0.591
	IDBE	61.60 ± 19.09	0.953	15	0.566
	Control	66.80 ± 41.86	0.969	15	0.845
EET	CSE	58.33 ± 21.64	0.973	15	0.899
	IDBE	63.73 ± 21.98	0.926	15	0.240
	Control	58.87 ± 13.27	0.960	15	0.690
SBTLt	CSE	60.93 ± 17.84	0.969	15	0.843
	IDBE	54.73 ± 12.17	0.962	15	0.720
	Control	64.24 ± 22.98	0.957	15	0.648
SBTRt	CSE	59.40 ± 19.85	0.983	15	0.985
	IDBE	61.87 ± 21.53	0.954	15	0.596
	Control	60.80 ± 17.47	0.943	15	0.416

Values are mean values ± standard deviations (SD); BMI—body mass index; CSE—core strengthening exercise; statistics—*t*-value of *t*-test; df—degree of freedom; *p*-value—level of significance; *p* insignificant at >0.05; CI—confidence interval; VAS—visual analog scale; ODI—Oswestry disability index; FET—flexors endurance test; EET—extensors endurance test; SBTLt: side bridge test (left); SBTRt—side bridge test (right).

**Table 2:** The tests of between-subjects’ effects within the participants’ treatment group, using a univariate ANCOVA test (*N* = 45; Missing = 0).

Post-Test Scores for Dependent Variables	Type-III Sum of Squares	df	Mean Square	F-Value	*p*-Value	η^2^
VAS	44.854	2	22.427	53.537	0.001 *	0.748 ˆ
ODI	7253.599	2	3626.799	194.512	0.001 *	0.915 ˆ
FET	15,950.781	2	7975.391	95.060	0.001 *	0.841 ˆ
EET	15,263.402	2	7631.701	38.345	0.001 *	0.681 ˆ
SBTLt	19,307.607	2	9653.803	142.225	0.001 *	0.888 ˆ
SBTRt	18,821.824	2	9410.912	127.420	0.001 *	0.876 ˆ

*—Significant value if *p* < 0.05; ˆ—large effect size if partial eta-squared (η^2^) value >0.6; CSE—core-strengthening exercise; df—degree of freedom; VAS—visual analog scale; ODI—Oswestry disability index; FET—flexors endurance test; EET—extensors endurance test; SBTLt—side bridges test (left); SBTRt—side bridges test (right).

**Table 3:** Pair-wise comparison of post-test scores (at six weeks) for the outcomes pain (VAS), disability (ODI), and multiple endurance tests (FET, EET, SBTLt, and SBTRt) between groups using the Bonferroni multiple comparison test (*N* = 45; Missing = 0).

Post-Test Scores for Dependent Variables	Treatment Groups	Mean Differences (∆MD ± SE)	*p*-Value	95% Confidence Interval for Difference	
				Lower Bound	Upper Bound
VAS	CSE vs. IDBE	−0.582 ± 0.257	0.088	−1.227	0.062
	CSE vs. Control	−2.762 ± 0.274	0.001 *	−3.451	−2.072
	IDBE vs Control	−2.179 ± 0.281	0.001 *	−2.885	−1.473
ODI	CSE vs. IDBE	−9.378 ± 1.712	0.001 *	−13.678	−5.079
	CSE vs. Control	−35.563 ± 1.831	0.001 *	−40.161	−30.964
	IDBE vs Control	−26.184 ± 1.875	0.001 *	−30.893	−21.475
FET	CSE vs. IDBE	12.621 ± 3.632	0.004 *	3.501	21.741
	CSE vs. Control	52.468 ± 3.885	0.001 *	42.714	62.222
	IDBE vs Control	39.847 ± 3.978	0.001 *	29.858	49.836
EET	CSE vs. IDBE	−1.113 ± 5.594	1.000	−15.159	12.934
	CSE vs. Control	46.676 ± 5.983	0.001 *	31.453	61.500
	IDBE vs Control	47.589 ± 6.127	0.001 *	32.204	62.974
SBTLt	CSE vs. IDBE	11.222 ± 3.267	0.005 *	3.019	19.425
	CSE vs. Control	57.066 ± 3.494	0.001 *	48.293	65.840
	IDBE vs Control	45.844 ± 3.578	0.001 *	36.859	54.829
SBTRt	CSE vs. IDBE	8.644 ± 3.408	0.047 *	0.087	17.201
	CSE vs. Control	55.625 ± −3.645	0.001 *	46.473	64.777
	IDBE vs Control	46.981 ± 3.732	0.001 *	37.609	56.354

*—Significant value if *p* < 0.05; VAS—visual analog scale; ODI—Oswestry disability index; CSE—core-strengthening exercise; df—degree of freedom; ∆MD—mean difference; SE—standard error; VAS—visual analog scale; ODI—Oswestry disability index; FET—flexors endurance test; EET—extensors endurance test; SBTLt—side bridges test (left); SBTRt—side bridges test (right).

**Table 4:** Correlation between pain intensity (VAS), functional disability (ODI), and multiple muscle endurance (FET, EET, SBTLt, and SBTRt) at baseline scores using Pearson’s correlation coefficient test (95% confidence interval, *N* = 45).

Variables (*N* = 45)	VAS r (*p*) Value	ODI r (*p*) Value	FET r (*p*) Value	EET r (*p*) Value	SBTLt r (*p*) Value	SBTRt r (*p*) Value
VAS	1	0.937 (0.001 **)	−0.803 (0.001 **)	−0.849 (0.001 **)	−0.819 (0.001 **)	−0.910 (0.001 **)
ODI	0.937 (0.001 **)	1	−0.758 (0.001 **)	−0.841 (0.001 **)	−0.821 (0.001 **)	−0.908 (0.001 **)
FET	−0.803 (0.001 **)	−0.758 (0.001 **)	1	0.872 (0.001 **)	0.772 (0.001 **)	0.816 (0.001 **)
EET	−0.849 (0.001 **)	−0.841 (0.001)	0.872 (0.001 **)	1	0.897 (0.001 **)	0.898 (0.001 **)
SBTLt	−0.819 (0.001)	−0.821 (0.001)	0.772 (0.001 **)	0.897 (0.001 **)	1	0.849 (0.001 **)
SBTRt	−0.910 (0.001)	−0.908 (0.001)	0.816 (0.001 **)	0.898 (0.001 **)	0.849 (0.001 **)	1

** Highly significant value (2-tailed), if *p* ≤ 0.01; *N*—total number of participants from CSE, IDBE, and Control groups; r—Pearson’s correlation coefficient value; VAS—visual analog scale; ODI—Oswestry disability index; FET—flexors endurance test; EET—extensors endurance test; SBTLt—side bridge test (left); SBTRt—side bridge test (right).

## Data Availability

The data that supports the study’s results will be available upon a reasonable request from the corresponding author.
